# Effects of natural childbirth preparation versus standard antenatal education on epidural rates, experience of childbirth and parental stress in mothers and fathers: a randomised controlled multicentre trial

**DOI:** 10.1111/j.1471-0528.2009.02144.x

**Published:** 2009-05-27

**Authors:** M Bergström, H Kieler, U Waldenström

**Affiliations:** a Department of Woman and Child Health, Retsius väg 13, Karolinska InstitutetStockholm; b Centre for Pharmacoepidemiology (CPE), Department of Medicine, Solna, Karolinska InstitutetStockholm, Sweden

**Keywords:** Antenatal education, childbirth experience, parenthood, pregnancy, psychoprophylaxis

## Abstract

**Objective:**

To examine the effects of antenatal education focussing on natural childbirth preparation with psychoprophylactic training versus standard antenatal education on the use of epidural analgesia, experience of childbirth and parental stress in first-time mothers and fathers.

**Design:**

Randomised controlled multicentre trial.

**Setting:**

Fifteen antenatal clinics in Sweden between January 2006 and May 2007.

**Sample:**

A total of 1087 nulliparous women and 1064 of their partners.

**Methods:**

*Natural* group: Antenatal education focussing on natural childbirth preparation with training in breathing and relaxation techniques (psychoprophylaxis). *Standard care* group*:* Standard antenatal education focussing on both childbirth and parenthood, without psychoprophylactic training. Both groups: Four 2-hour sessions in groups of 12 participants during third trimester of pregnancy and one follow-up after delivery.

**Main outcome measures:**

Epidural analgesia during labour, experience of childbirth as measured by the Wijma Delivery Experience Questionnaire (B), and parental stress measured by the Swedish Parenthood Stress Questionnaire.

**Results:**

The epidural rate was 52% in both groups. There were no statistically significant differences in the experience of childbirth or parental stress between the randomised groups, either in women or men. Seventy percent of the women in the *Natural* group reported having used psychoprophylaxis during labour. A minority in the *Standard care* group (37%) had also used this method, but subgroup analysis where these women were excluded did not change the principal findings.

**Conclusion:**

Natural childbirth preparation including training in breathing and relaxation did not decrease the use of epidural analgesia during labour, nor did it improve the birth experience or affect parental stress in early parenthood in nulliparous women and men, compared with a standard form of antenatal education.

## Introduction

Antenatal education has been offered to pregnant women over a long period of time in most high income countries and more recently also to expectant fathers. Such education may be a component of routine antenatal care within a country’s healthcare system, or organised by different stakeholders outside the system. In Sweden, 93% of the nulliparous women and 84% of their partners attended antenatal education classes in year 2000, most of which were organised within the public healthcare system with midwives practicing in the antenatal clinics as educators.^1^

The content of antenatal education in Western societies has shifted over time. In the 1940s, the focus was on physical exercise as a way to remain fit in spite of the physical changes of pregnancy. The British obstetrician Dick-Read focused on labour pains and how these were affected by muscle tension triggered by fear.^2^ By giving information about the process of labour and practical training in relaxation, fear and tension would be reduced and as a consequence also labour pain. About the same time, the French obstetrician Fernand Lamaze introduced psychoprophylaxis.^3^ The method was developed in Russia and emphasised relaxation as a conditioned response to labour contractions, coupled with a variety of patterned breathing techniques designed to improve oxygenation and interfere with the transmission of pain signals from the uterus to the brain.^4^ During the 1970’s this method was spread more widely in many Western societies, but in Sweden, like in other countries, it more or less lost its popularity two decades later.^5^,^6^

With the development of obstetric care, information about pharmacological methods of pain relief and medical interventions now constitute a large component of antenatal education. Also parenthood issues play a more important role, partly because of the increased involvement of expectant fathers.^1^,^7^ Traditional teaching techniques have been replaced by group discussions where the participants may raise their own questions.^8^ Interestingly although, psychoprophylaxis is now being reintroduced in Sweden and the method is being taught to a new generation of midwives.^9^

Antenatal education has been sensitive to opinions and trends and has undergone dramatic changes without us knowing much about its effects on relevant outcomes. It represents considerable costs,^8^ but is poorly evaluated.^10^ The aims are often broad and general, such as preparation for childbirth and parenthood,^11^,^12^ both outcomes that may be difficult to measure, thus making evaluations scarce.

In this randomised controlled trial we were interested to know the effect of antenatal group education on the three outcomes we found most relevant in relation to the aims: Labour pain expressed as a need for epidural analgesia, overall experience of childbirth and experience of parental stress in early parenthood.

As antenatal classes are attended by most expectant first-time parents we could not compare current form of education with no education at all. Therefore, we created a *Natural* model focussing on preparation for childbirth only, including training in psychoprophylaxis. This model was compared with a *Standard care* model, which in accordance with clinical practice in Sweden at the onset of the trial allocated equal time to preparation for childbirth and parenthood. This model did not include any psychoprophylactic practice. The principal difference between the two models was that the first prepared for natural childbirth and the second for parenthood as well as childbirth. We hypothesised that participants in the *Natural* group would have lower rates of epidural analgesia, a more positive overall experience of childbirth, but a higher degree of parental stress compared with the *Standard care* group.

## Methods

We conducted a randomised controlled trial in which pregnant women with their partners, as well as the educators performing the interventions, were allocated to the *Natural* model or the *Standard care* model. The educators were randomised individually to lead groups according to either model during the entire study period. The pregnant women and their partners were randomised in groups of 12 persons, or six couples. Both models of education were given at each participating clinic.

### Educators

All antenatal care midwives in Sweden were informed about the study either by their regional midwifery coordinator or an advertisement in the *Swedish Journal of Midwifery*. At least two midwives from each clinic, willing to be randomised to lead either model of education, were required. From Sweden’s around 500 antenatal clinics, 43 midwives from 16 clinics participated at the commencement of the trial. The participating clinics had a representative geographical distribution, including both urban and rural areas. During the trial, eight educators withdrew for medical reasons or changes in employment conditions. Three of these were from the *Natural* and five from the *Standard care* group. Two of the *Standard care* midwives who withdrew were replaced by colleagues. These received an introduction to the trial and the model of education with similar content as that given to the other midwives. The 35 midwives in the study completed one to ten education groups from January 2006 to May 2007 with a median number of five groups. The educators had a mean of 11 years of previous experience of childbirth education. No educational groups outside of the trial were held by the educators during the study period.

Before onset of the trial all educators participated in a 1-day workshop about the methodology of randomised controlled trials and the importance of adhering to the allocated model without discussing and sharing the content with their colleagues. In addition, the educators of the *Natural* model were trained to lead the new model during a 2-day workshop. Two 1-day follow-up workshops for all participating educators were organised during the course of the trial.

### Participants

**Figure 1:**
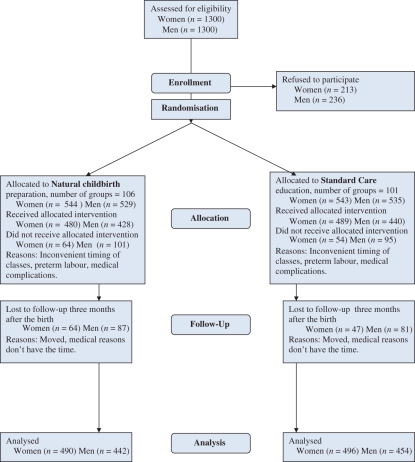
Flowchart of participants flow from recruitment to follow-up at 3 months postpartum.

Women were eligible for the study if they were nulliparous, Swedish-speaking and attending any of the participating clinics. No specific inclusion criteria were defined for their partners. Eligible women and their partners were informed about the study by their antenatal care midwife at approximately 19 gestational weeks. Written information including a baseline questionnaire was handed out after obtained consent and inclusion in the trial took place when the completed baseline questionnaire was returned to the research group. Figure 1 shows the trial profile including losses to follow up. Altogether 1087 nulliparous women and 1064 of their partners were recruited from October 2005 to February 2007. The recruiting midwives estimated the number of eligible women to approximately 1300. The most common reason for declining participation was preference for attending open lectures rather than educational groups. The participating women and their partners were randomised into 207 groups: 106 *Natural* groups and 101 *Standard care* groups, with a median number of 12 participants per group. A total of 986 (91%) women and 896 (84%) men completed the follow-up questionnaire 3 months after birth. The response rate was similar for both arms. The follow-up questions were answered through the website by 242 women and 186 men, whereas all others completed the paper version.

### Interventions

**Figure 2:**
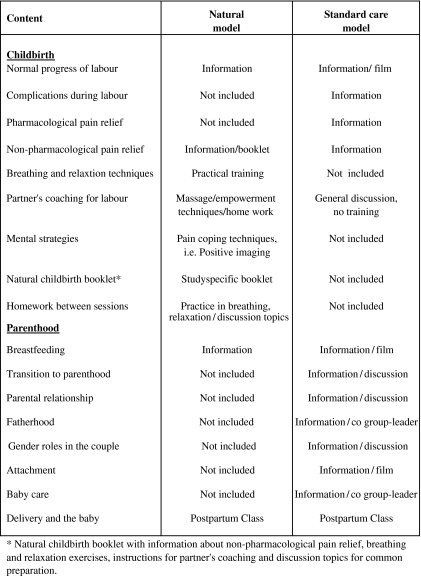
Description of the two models of antenatal education.

The two antenatal education models had the same structure but different content. Both models included four 2-hour sessions during pregnancy and one follow-up session within 10 weeks after delivery. The classes commenced during the third trimester of pregnancy. The size of each group was 12 persons or six couples. The content of the models is shown in Figure 2.

The *Natural* model was manual based. Focus was on preparation for natural childbirth. Information was given about non-pharmacological methods for pain relief and the partner’s role as a coach during labour. In each session, 30 minutes were spent on practical training in breathing, relaxation and massage techniques. Psychoprophylactic training between sessions was encouraged and a booklet to facilitate homework was distributed. The attitude of the educator was encouraged to be in favour of natural birth. Information about breastfeeding was provided but no other postnatal issues were addressed. If possible one of the sessions could include a visit to the delivery ward.

In the *Standard care* model, equal time was allocated to information and discussion about childbirth and parenthood issues to reflect the content of antenatal education as provided by antenatal clinics in Sweden. Within these limits the teaching methods of the *Standard care* groups could vary. The educators in this model were free to present films, arrange visits to the delivery ward and to invite external experts as co-educators according to local practice. No information about breathing, relaxation or other specific techniques for coping with labour pain was included.

### Outcomes and data collection

The outcomes of the trial were epidural rates, experience of childbirth and parental stress in early parenthood. Data were collected by two questionnaires: at baseline before randomisation and 3 months after birth. A postal card was sent to the parents asking them to complete the second questionnaire on the web-based homepage. A letter of reminder, including a paper version of the questionnaires, was sent 1 week later and a second reminder after two more weeks. A telephone call to non-responders was made after another 2 weeks.

The baseline questionnaire included questions about sociodemographic background, expectations and attitudes to antenatal education, childbirth and early parenthood. The follow-up questionnaire asked about experiences of childbirth and early parenthood and a few other questions not reported in this paper. The questions about antenatal education were designed specifically for the study.

We used the Wijma Delivery Expectancy/Experience Questionnaire, version A and B, to measure expectations (baseline questionnaire) and experience (follow-up questionnaire) of childbirth.^13^ These scales were developed to measure pre- and postnatal fear of childbirth, with high validity and reliability. Both versions have 33 items with six-point response scales covering various feelings and cognitive appraisal of childbirth. The fathers’ expectations and experiences were measured by 25 items from the W-DEQ. Eight items were excluded, as they were found irrelevant for fathers. A high score indicates a less satisfying experience. Maximum score for women is 165 and for men 125. The mean score on the 33-item version was around 50 in nulliparous and parous women in another study.^14^ This would correspond to a mean of 38 on the 25-item version. In addition to the W-DEQ, we asked both women and men during pregnancy about their expectations on the approaching birth, and after birth about their overall experience of childbirth, using a question with 5 response alternatives ranging from “very positive” to “very negative”.^15^

Memory of labour pain was rated by the women on an eight-point Likert scale, where 0 indicated ‘no pain at all’ and seven ‘worst imaginable pain’. A similar numerical scale has been used in several studies investigating memory for labour pain.^6^,^16^,^17^ Parental stress was measured in both men and women by the Swedish Parenthood Stress Questionnaire (SPSQ), which is an adapted and modified version of the American ‘Parenting Stress Index’.^18^–^21^ In Swedish samples, the SPSQ has been shown to have a stable factor pattern constituting the subscales incompetence, role restriction, social isolation, spouse relationship problems and health problems. On this 34-item scale, a high score indicates higher perceived stress in parenthood. Mean total score in mothers of 6-months-old babies was 2.14 in an earlier study.^19^ The validity and reliability of the SPSQ have been found to be good.^19^,^21^

Adherence to the interventions was measured by an internet-based process evaluation questionnaire filled in by the educators after each completed study group. Detailed information on class content, teaching styles, group atmosphere and the educator’s assessment of her own performance was obtained.

Information about the interventions was also obtained from the participants in the follow-up questionnaire. The study participants could tick from a list which topics that had been discussed and indicate how much time that was allocated to each topic. They could also indicate if there had been film presentations, any co group-leaders or a visit at the delivery ward.

### Sample size estimation and statistical analysis

To detect a reduction in epidural rates from estimated 50% in the *Standard care* group to 40% in the *Natural* group a total sample of 776 women was required (80% power; *P < 0.05*). This sample size would also allow the detection of a difference of 0.27 in mean scores on the Swedish Parental Stress Questionnaire (80% power; *P < 0.05*). No estimates were made regarding the experience of childbirth because of lack of reference data for the *Standard Care* group. When adjusting for possible cluster effects (intra-class correlation coefficient = 0.02, variation in cluster size 0–5, inflation factor of 1.125), the required sample size was estimated to 916 women; 458 in each arm.^22^,^23^ No power calculation was made on the experience of childbirth and parental stress among fathers because of lack of reliable estimates in the *Standard care* group.

We used an internet-based system for registration, randomisation and process evaluation (http://www.medscinet.com/tuff, MedSciNet AB, Stockholm, Sweden). The groups were formed by midwives listing the participants in this computer system according to their estimated date of delivery (EDD). A group consisted of women with similar EDDs and their partners. When a group included twelve individuals the entire group was randomised to one of the two models. The randomisation was conducted by the computerised algorithm with two priorities: Stratification by (1) equal number of participants per model in all clinics taken together and (2) balancing the numbers of each model within the respective clinic.

Data were analysed according to the intention to treat principle and blinding to group allocation was not possible. As some women in the *Standard care* groups attended psychoprophylaxis classes outside of the trial or practised psychoprophylaxis at home we also performed additional analyses where these women were excluded. A comparison on principal outcomes between women who used psychoprophylaxis during labour and those who did not was also performed disregarding the randomised groups.

We compared continuous variables by *t* tests and categorical data by chi square tests. Data are presented as mean or median, together with standard deviations (SD) or range and as differences of mean or relative risks (RR) with 95% confidence intervals (CI). Statistical analyses were performed in spss 15.0 (SPSS, Chicago, IL, USA). All data were kept confidential and analyses were not performed until completion of the study. Blinding to group allocation was maintained during data entry but was not possible during the analyses.

## Results

### Characteristics

Baseline characteristics of women and men were similar in both groups (Table 1). More women in the *Standard care* group had, before randomisation, a positive attitude to psychoprophylaxis during labour than women in the *Natural* group, and women and men in the *Natural* group had a more positive attitude to epidural analgesia during labour. In the *Natural* group, 24% of the women and 16% of the men had a positive attitude to both epidural analgesia and psychoprophylaxis as did 25% of the women and 17% of the men in the *Standard care* group.

**Table 1:** Baseline characteristics of women and men in the *Natural* and *Standard care* groups

Characteristics	Women		Men	
	Natural	Standard	Natural	Standard
	*n*= 544	*n*= 543	*n*= 529	*n*= 534
	**Mean (range)/*n* (%)**	Mean (range)/*n* (%)	Mean (range)/*n* (%)	Mean (range)/*n* (%)
**Mean age (range)**	28.8 (18–46)	28.6 (17–44)	31.4 (19–60)	31.5 (18–62)
**Mean BMI prior to pregnancy (range)**	23.3 (16.0–48.9)	23.6 (15.8–47.6)		
**Expecting first baby, *n* (%)**	544 (100)	543 (100)	480 (91)	480 (90)
**Civil status: married or cohabiting, *n* (%)**	527 (97)	522 (96)	515 (97)	520 (98)
**Born in Sweden, *n* (%)**	506 (93)	498 (92)	492 (93)	502 (94)
**Highest education, *n* (%)**				
Elementary school	20 (4)	21 (4)	29 (6)	29 (6)
High school	238 (44)	251 (46)	283 (54)	306 (58)
College or university	283 (52)	270 (50)	213 (41)	197 (37)
**Positive attitude to psychoprophylaxis during labour, *n* (%)**	314 (58)	341 (63)	246 (47)	250 (47)
**Positive attitude to epidural analgesia during labour, *n* (%)**	214 (39)	201 (37)	169 (32)	161 (30)
**Positive attitude to both epidural and psychoprophylaxis, *n* (%)**	128 (24)	136 (25)	86 (16)	89 (17)

### Adherence to the interventions

Both models of education included 8 hours of antenatal preparation. According to the web-based process evaluation conducted by the educators after each completed group the *Natural* group spent a mean of 5.8 hours per group on labour and birth issues, of which 2.6 hours were allocated to psychoprophylaxis. Postnatal issues were addressed during mean 1.7 hours and were primarily about breastfeeding. The remaining half hour was spent on discussion of miscellaneous topics raised by the group members. Nine percent of the women (*n*= 43) and 10% of the men (*n*= 45) in this group had visited the delivery ward as part of the education.

In the *Standard care* group, a mean of 3.9 hours had been allocated to childbirth preparation and 3.5 hours to issues about the newborn and the postnatal period. The remaining half hour was spent on discussion of topics raised by the group members. No practical training in psychoprophylaxis was included. Film presentations were common: 95% (*n*= 465) of the women and 90% (*n*= 401) of the men reported having watched at least one film, mostly about childbirth. The most common co group-leader was a man talking about fatherhood during one of the sessions and this was reported by 10% of the participants (women = 50, men = 47). Twenty-one percent (*n*= 105) of the women and 20% (*n*= 91) of the men in this group visited the delivery ward within the frame of the education.

### Psychoprophylactic practice

In the *Natural* group, 85% (*n*= 411) of the women and 73% (*n*= 315) of the men practised psychoprophylaxis at home during pregnancy and 70% (*n*= 331) of the women said they had used the technique during labour. In the *Standard care* group, 8% (*n*= 37) of the women and 7% (*n*= 30) of the men had attended private classes in psychoprophylaxis outside of the trial. The method was practised at home by 45% (*n*= 219) of the women and 21% (*n*= 95) of the men in this group and 37% (*n*= 179) of the women said they had used psychoprophylaxis during labour.

### Outcomes of the intention to treat analysis

As illustrated in Table 2, the experiences of childbirth and parental stress were similar in the two groups. The epidural rate was 52% in both groups. In both groups, 66% had a normal vaginal delivery and the mean (SD) duration of labour was 11 (9.9) hours. The Caesarean section rate was 20% and 21.5% in the *Natural* and *Standard care* group, respectively, and the rate of instrumental vaginal delivery 14% and 12%.

**Table 2:** Mode of delivery, epidural analgesia, experience of childbirth and parental stress in women and men in the *Natural* and *Standard care* groups

Measure	Women				Men			
	Natural	Standard	RR/Mean difference	*p*	Natural	Standard	RR/Mean difference	*p*
	*n*= 484	*n*= 493	(95% CI)		*n*= 413	*n*= 420	(95% CI)	
**Mode of delivery, *n* (%)**								
Spontaneous vaginal	321 (66)	327 (66)	1.0 (0.9 to 1.1)	1.0				
Instrumental	67 (14)	60 (12)	1.1 (0.8 to 1.6)	0.4				
Elective Caesarean	29 (6)	31 (6)	0.9 (0.6 to 1.6)	0.8				
Emergency Caesarean	67 (14)	75 (15)	0.9 (0.7 to 1.2)	0.5				
**Epidural analgesia, *n* (%)**	247 (52)	252 (52)	1.0 (0.9 to 1.1)	0.9				
Memory of labour pain*, mean (SD)	4.9 (1.8)	4.9 (1.8)	0 (−0.2 to 0.3)	0.7				
**Experience of childbirth**								
W-DEQ B**, mean (SD)	49.6 (26)	50.1 (25)	−0.5 (−3.2 to 4.1)	1.0	36.6 (16)	38.2 (18)	−1.6 (−0.7 to 4.0)	0.1
‘Very negative’ and ‘negative, *n* (%)	42 (9)	49 (10)	0.9 (0.6 to 1.3)	0.5	16 (4)	24 (5)	0.7 (0.4 to 1.3)	0.2
**Experience of parenthood**								
Total SPSQ***, mean (SD)	2.3 (0.5)	2.3 (0.5)	0 (−0.0 to 0.1)	0.6	2.2 (0.4)	2.3 (0.5)	−0.1 (0.0 to 0.1)	0.4
‘Very positive’, *n* (%)	368 (77)	370 (76)	1.0 (0.9 to 1.1)	0.8	343 (80)	347 (78)	1.0 (0.9 to 1.1)	0.5

Comparisons are expressed as relative risks (RR) for nominal variables and as differences of means for continuous variables together with 95% confidence intervals (95% CI).

* Likert scale ranging from ‘0 = no pain’ to ‘7 = worst pain imaginable’.

** Items range from 0 to 5 with 5 as most negative. Women: 33 items, maximum total score 165. Men: 25 items, maximum total score 125. Cronbach’s alpha: Women 0.94; Men 0.90.

*** Thirty-four items, range 1–5 with 5 as most negative. Cronbach’s alpha: Women 0.88; Men 0.87.

The women rated childbirth as a ‘very negative’ or ‘negative’ experience to a higher extent than the men, but there were no statistically significant differences between the trial groups. Memory of labour pain was also similar between women in the two groups. A large majority of women and men in both groups said parenthood was a very positive experience at 3 months after the delivery and there were no statistically significant differences between the groups*.*

### Subgroup analysis

In the subgroup analyses (Table 3), we performed two analyses on outcomes related to childbirth where some women from the *Standard care* group were excluded. In the first analysis, the women in the *Standard care* group who had attended private psychoprophylaxis classes outside the trial (*n*= 37) were excluded. In the second analysis, the women in the *Standard care* group who had practised psychoprophylaxis at home during pregnancy (*n*= 219) were excluded. In a third analysis, we compared women who used psychoprophylaxis during labour with those who did not, on the same outcomes, disregarding the randomised groups. We found no statistically significant differences between the groups in these analyses.

**Table 3:** Epidural analgesia and experience of childbirth in women when controlling for use of psychoprophylaxis in the Standard care group by subgroup analyses

Subgroups	Epidural			Experience of childbirth W-DEQ B*			Experience of childbirth ‘Very negative’ and ‘negative’		
	*n* (%)	RR (95% CI)	*p* (χ^2^)	Mean (SD)	Mean difference (95% CI)	*p*	*n* (%)	RR (95% CI)	*p* (χ^2^)
**37 women in Standard care group excluded who had attended psychoprophylaxis classes**									
Natural, *n*= 473	247 (52)	1.0 (0.9 to 1.1)	0.8	49.6 (26)	−0.8 (−3.0 to 4.4)	0.7	42 (9)	0.9 (0.6 to 1.3)	0.5
Standard care, *n*= 449	241 (54)			50.4 (25)			46 (10)		
**219 women in Standard care group excluded who had practiced psychoprophylaxis at home**									
Natural, *n*= 473	247 (52)	0.9 (0.8 to 1.1)	0.4	49.6 (26)	−0.6 (−3.7 to 4.8)	0.8	42 (9)	0.9 (0.6 to 1.4)	0.7
Standard care, *n*= 267	149 (56)			50.2 (24)			26 (10)		
**Women who used psychoprophylaxis during labour versus those who did not, randomised groups amalgamated**									
Psychoprophylaxis, *n*= 510	271 (53)	1.0 (0.9 to 1.2)	0.7	50.0 (26)	−0.4 (−3.2 to 4.1)	0.8	40 (8)	0.7 (0.5 to 1.0)	0.08
No Psychoprophylaxis, *n*= 443	230 (52)			49.6 (25)			49 (11)		

Comparisons are expressed as relative risks (RR) for nominal variables and as differences of means for continuous variables together with 95% confidence intervals (95% CI).

* Thirty-three items ranging from 0 to 5 with 5 as most negative. Maximum total score 165.

## Discussion

This randomised controlled trial compared two models of antenatal group education: One that focused primarily on preparation for natural childbirth with practical training in psychoprophylaxis, the *Natural* group, and the other reflecting standard childbirth and parenthood education as provided within the Swedish antenatal care program, *Standard care* group. We found no statistically significant differences between the groups in rates of epidural analgesia, satisfaction with the childbirth experience or parental stress at 3 months postpartum. The findings suggest that psychoprophylactic training as practised in the *Natural group* does not reduce the need for epidural analgesia or improve the childbirth experience, and parenthood preparation as practised in the *Standard care* group does not reduce experienced stress in early parenthood. However, the lack of statistically significant differences in the three outcomes could also be related to insufficient differences between the two models, or the choice of outcome measures.

### Differences between the models

The two most noticeable differences between the models were the psychoprophylaxis component in the *Natural* group and the parenthood component in the *Standard care* group. It was obvious that the psychoprophylaxis component affected women’s and men’s behaviour as many more in the *Natural* group practised psychoprophylaxis at home during pregnancy and also used the method during labour compared with the *Standard care* group. However, the finding that some women in the *Standard care* group also used the method during labour may have diluted the effect of the antenatal exposure. As both models of education were given at each of the participating clinics one may suspect that the use of psychoprophylaxis in the *Standard care* group could be because of contamination between the models. However, the importance of avoiding cross-over effects was discussed in detail with the educators before starting the trial and the follow-up sessions reassured us that all educators adhered to the protocol. The process evaluation also showed that there was no reason to worry for this reason, as the reports from the educators were similar to those of the participants. Therefore, we believe that the use of psychoprophylaxis in the *Standard care* group is principally explained by influences from outside of the trial, because of the increasing popularity of psychoprophylaxis in Sweden. But it is also possible that participation in the trial may have increased awareness of the method.

### Outcome measures

The principal outcomes of the trial were chosen to reflect the aims of childbirth education today. We chose epidural analgesia as one of the main outcomes, assuming that it would reflect experience of in-labour pain. We also measured memory of labour pain at 3 months postpartum. As none of these outcomes differed between the groups we conclude that women used psychoprophylaxis as a complement rather than as a replacement of epidural analgesia. This interpretation is supported by our baseline data, which shows that a substantial proportion of the women and men who had a positive attitude to psychoprophylaxis in mid-pregnancy also had a positive attitude to epidural analgesia.

The epidural rate in women who gave birth vaginally was 49% in both groups, a figure comparable with the national statistics for nulliparous women, which was 46%.^25^ This finding shows that neither model reduced the need for epidural analgesia. It may be argued that participation in antenatal education *per se* instead increase the epidural rate as previously reported in an observational study comparing attendees with non-attendees.^1^

The overall experience of childbirth is a complex outcome as it includes the experience of hours of pain and hard work, as well as the encounter with the newborn baby. The W-DEQ is a comprehensive instrument, which aims at capturing both feelings during labour, such as pain, fear and confidence, but also the assessment of the total experience of the birth.^13^ Besides using this instrument, we asked women and men to assess their experience of childbirth by a single item question and the women also rated the intensity of labour pain as they remembered it at 3 months postpartum. We believe we have investigated the overall experience of childbirth in the best possible way when quantitative measures are necessary because of a large number of participants. Our findings are supported by research from observational studies where antenatal education had no positive effects on the risk of being dissatisfied with the childbirth experience.^15^ However, we cannot exclude the possibility that the antenatal education models effected aspects of the birth experience that were not captured in this study.

Measuring the effect of antenatal education on parents’ experiences of parenthood was an even greater challenge. Still, this is an important outcome since parental issues have become a more important component of antenatal education over the recent years. It has been assumed that preparation for parenthood would be of interest to expectant fathers in particular.^25^,^26^ The instrument we found most appropriate was the SPSQ^19^–^21^ as it is a well-validated and comprehensive instrument that addresses different aspects of parenthood, such as feelings of incompetence, role restriction, social isolation, spouse relationship problems and health problems. Based on our results we question whether parenthood preparation, apart from information about breastfeeding, should be included in antenatal education. Women as well as men may have difficulties seeing beyond the challenge of childbirth during pregnancy.

### Subgroup analyses

As a small number of women in the *Standard care* group had attended antenatal classes in psychoprophylaxis outside of the trial and had practised the method at home during pregnancy we conducted additional analyses in which we excluded these women. The results of these analyses confirmed that the psychoprophylaxis component of the *Natural* model had no effect on the studied outcomes.

When we compared the women in both groups who used psychoprophylaxis during labour with those who did not, we found that psychoprophylaxis had no effect on use of epidural analgesia or experience of childbirth. This conclusion, however, needs to be confirmed by randomised controlled trials. Women who choose to use psychoprophylaxis may differ from those who do not use it, regarding background characteristics and attitudes.

### Methodological issues

To our knowledge this is the first large randomised controlled trial of antenatal group education that also includes the men and study relevant outcomes, such as experience of childbirth and early parenthood. We would ideally have conducted a study where half of the participants had been randomised to no education. This was, however, believed to be impossible in a context where antenatal education is an established component of antenatal care and believed to be helpful by pregnant women and their partners, as well as by most professionals in the field.

The use of epidural analgesia was measured through women’s own reporting 3 months after the birth. Self-reported use of epidural has previously been found reliable when compared with data from the Swedish Medical Birth Register.^6^ We have not investigated whether there were differences between the groups regarding the time-point during labour when epidural analgesia was administered, as we did not access such information.

We have analysed data of individuals in spite of the fact that exposures was given to groups of individuals. This increases the risk of cluster effects, i.e., that certain common attitudes are adapted within a group or that some individuals affect the group climate and the participants. We had therefore planned to adjust for cluster effects in the analyses, but because of the minimal differences between the groups this was not necessary.

To assess how representative the participants were for childbearing women and men in general, we made comparisons with the total population of women giving birth in Sweden. We found that maternal age was similar to Swedish first-time mothers in general,^24^ but that women and men with the lowest level of education were slightly underrepresented as were women born outside of Sweden. When comparing our sample with a representative sample of 1101 pregnant Swedish speaking nulliparous women who attended or did not attend antenatal education classes we found about the same percentage of married or cohabiting women and the same percentage of low educated women as in the attendees. Women with the lowest level of education and with another native language are less inclined to participate in antenatal group education.^1^ Altogether, we consider our sample representative for those normally reached by antenatal education in Sweden.

## Conclusion

Natural childbirth preparation including psychoprophylactic training does not reduce the need for epidural analgesia or improve the birth experience, when compared with antenatal education where childbirth issues are allocated less time and only addressed in theory. Parental stress in mothers and fathers in early parenthood may not be affected by addressing parental issues in general antenatal education.
